# Real-time quantitative PCR for analysis of candidate fungal biopesticides against malaria: Technique validation and first applications

**DOI:** 10.1016/j.jip.2009.01.006

**Published:** 2009-03

**Authors:** Andrew S. Bell, Simon Blanford, Nina Jenkins, Matthew B. Thomas, Andrew F. Read

**Affiliations:** a School of Biological Sciences, University of Edinburgh, West Mains Road, Edinburgh EH9 3JT, UK; b Centre for Infectious Disease Dynamics, Departments of Biology and Entomology, Pennsylvania State University, University Park, PA 16802, USA; c CSIRO Entomology, GPO Box 1700, Canberra ACT 2601, Australia

**Keywords:** Real-time quantitative PCR assays, Fungal biopesticides, Malaria, *Plasmodium chabaudi*, *Beauveria bassiana*, *Metarhizium anisopliae*, *Anopheles stephensi*, Growth kinetics, Vector control

## Abstract

Recent research has indicated that fungal biopesticides could augment existing malaria vector control tools. Here we present a set of methodologies to monitor the *in vivo* kinetics of entomopathogenic fungi in *Anopheles* in the presence or absence of malaria parasites using quantitative real-time PCR. Three qPCR assays were successfully developed for counting fungal genomes: “specific” assays capable of distinguishing two well characterized fungal entomopathogens *Beauveria bassiana* isolate IMI391510 and *Metarhizium anisopliae* var. *acridum* isolate IMI330189, both of which have previously been shown to be virulent to *Anopheles* mosquitoes, and a “generic” fungal assay for determining any fungal burden. A fourth assay to *Plasmodium chabaudi* enabled quantification of co-infecting malarial parasites. All qPCR assays provide sensitive, target-specific, and robust quantification over a linear range of greater than five orders of magnitude (seven orders of magnitude for the fungal assays). *B. bassiana* growth within mosquitoes exposed to three different conidial challenge doses was monitored using the *B. bassiana*-specific assay and represents the first description of entomopathogenic fungal replication within an insect host. This revealed that, irrespective of challenge dose, after several days of relatively little replication, a sudden on-set of substantial nuclear division occurs, accompanied by physical fungal growth (hyphae) within the mosquito haemocoel shortly before death. Exposure to higher densities of conidia resulted in significantly greater pick-up by mosquitoes and to elevated fungal burdens at each time point sampled. High fungal burdens, comparable to those identified in cadavers, were attained more rapidly and mortalities occurred earlier post-exposure with increasing challenge dose. The lines of research made possible by the qPCR assays described here will contribute to optimization of fungal biopesticides against malaria and other vector-borne diseases.

## 1 Introduction

The evolution of insecticide resistance and possible environmental and human health risks increasingly challenge the otherwise successful use of chemical insecticides to control vector-borne diseases like malaria and dengue (Shiff, 2002). Recent research has raised the prospect that fungal biopesticides could augment existing vector control tools. These insecticides are based on oil-formulated spores of entomopathogenic fungi applied to surfaces on which adult mosquitoes will rest after blood feeding. Biopesticides for malaria control are still at an early research stage, but they very effectively block malaria transmission in the laboratory and can be delivered in African houses (Blanford et al., 2005; Scholte et al., 2005; Thomas and Read, 2007).

Research on insect fungal pathogens such as *Beauveria bassiana* and *Metarhizium anisopliae* has a longer history in the context of agricultural pests. Modern molecular techniques have enabled the characterization, detection and tracking of fungal isolates in the environment (e.g. Hegedus and Khachatourians, 1995; Castrillo et al., 2003; Hynes et al., 2006; Takatsuka, 2007), and the elucidation of mechanisms involved in host recognition and penetration, toxin production, and immune stimulation and evasion (e.g. St. Leger et al., 1996; Gillespie et al., 1998; Charnley, 2003; Ouedraogo et al., 2003; Dean et al., 2004, Wang and St. Leger, 2006). However, several determinants of fungal biopesticide efficacy remain inaccessible, particularly the factors influencing spore loads contacted by target insects and the subsequent kinetics of fungal growth within an insect. Progress on these issues requires determination of fungal load.

Standard approaches for assessing fungal load *in vivo* centre on visual quantification of blastospores and mycelial fragments in hemolymph, or of numbers of colony forming units (CFUs) in cultures of hemolymph or other body parts (Goettel and Inglis, 1997). These techniques have their limitations. For example, Ouedraogo et al. (2003) used measures of hyphal body concentration and CFUs from hemolymph samples to investigate the effects of temperature on the growth of *M. anisopliae* var. *acridum* in locusts. Hyphal bodies could not be detected microscopically until 3 days after inoculation, suggesting insensitivity at low fungal concentrations, but CFUs, only detectable from day 2, rapidly became too numerous to be counted on subsequent days, suggesting insensitivity at high fungal concentrations. More generally, the CFU technique requires a selective media (not necessarily available for all species), is unable to differentiate between colonies developing from a single cell vs. clumps of cells or mycelial fragments, and neither technique is able to differentiate between co-infecting strains of fungi.

Here we present a set of methodologies to monitor the *in vivo* kinetics of entomopathogenic fungi in *Anopheles* in the presence or absence of malaria parasites using quantitative real-time PCR. qPCR has been utilized to quantify, among others, *Aspergillus*, *Candida* and *Pneumocystis* fungi, with authors extolling its enhanced sensitivity, objectivity and speed (see Espy et al., 2006 for review). Indeed, Castrillo et al. (2008) recently used qPCR for determining the persistence of *B. bassiana* (strain GHA) sprayed on ash trees and leached onto soil. We sought to exploit this technology in the context of malaria control because there are a large range of questions relating to the lethal and sub-lethal effects of different fungal isolates on different mosquitoes, the interaction between co-infecting isolates and between fungi and malaria parasites, the effect of mosquito condition and environment on the outcome of infection, and influence of behavior and delivery systems on fungal infection. Here we illustrate how, by providing quantitative measures of fungal load/growth over time, the qPCR approach will enable thorough investigation of such questions in the future. We expect the techniques presented here will also be useful for biopesticides against other public health and agricultural pest problems.

## 2 Materials and methods

### 2.1 Overview

Fungal assays were designed for the quantification of the number of fungal genomes on and/or within mosquitoes which may also be infected with malaria parasites. Fungal kinetics within mosquitoes was defined in terms of nuclear division: the increase in fungal genome number with respect to time post-challenge. Counts were based on “conidial units”: a single unit being the number of copies of the target gene within a single conidium. We focused our work on the three fungal isolates which are currently the leading candidates for biopesticide control of malaria. For two of these, we developed assays which can distinguish two of these from any of the other three (hereafter call “specific” assays), and a single assay that could quantify any of the three (hereafter called the “generic” assay). “Specific” assays were designed to discriminate the target isolate from the other isolates utilized in the current study, so that in future studies, each isolate of interest can be quantitated in mixed infections. The general specificity of these assays was not tested because such global isolate specificity was not the aim: we are developing tools to work experimentally with the particular isolates that are current candidates for malaria control. This means that the utility of the *B. bassiana* isolate IMI391510 assay is likely to extend to other *B. bassiana* isolates that share sequence identity. Quantitation of malaria parasite load in fungal-infected mosquitoes will also be of frequent interest, and so we included a *Plasmodium* assay in our work, and checked for cross-reactivity. We primarily used *P. chabaudi* from laboratory mice, a rodent model of human malaria, but checked it would also work with other *Plasmodium* species likely be involved in subsequent developments of fungal biopesticides. Each fungal or *Plasmodium* assay was tested to ensure repeatability, linearity across the dynamic range and specificity to their targeted DNAs. We then used the *B. bassiana*-specific qPCR protocol to determine its growth kinetics in mosquitoes that had been exposed to three different challenge doses of conidia.

### 2.2 Mosquitoes and fungi

*Anopheles stephensi* larvae were reared under standard insectary conditions at 26 °C, 75% humidity and a 12L:12D photo-period. Eggs were placed in plastic trays (25 cm × 25 cm × 7 cm) filled with 1.5 l of distilled water. To reduce variation in adult size at emergence, larvae were reared at a fixed density of 400 per tray. Larvae were fed on *Liquifry* for 5 days and then on *TetraFin* fish flakes. From approximately two weeks after egg hatch pupae were collected daily and placed in emergence cages. The adults that emerged were fed *ad libitum* on a 10% glucose solution supplemented with 0.05% paraaminobenzoic acid (PABA). Adult female mosquitoes between 4 and 6 days old were equally distributed across all experimental cages.

Three mitosporic Ascomycete entomopathogenic fungi were used in this study; *B. bassiana* isolate IMI391510, *M. anisopliae* var. *anisopliae* isolate ICIPE30 and *M. anisopliae* var. *acridum* isolate IMI330189. Two of these isolates (IMI391510 and ICIPE30) have previously been shown to successfully infect *Anopheles* mosquitoes and to have malaria control potential (Blanford et al., 2005; Scholte et al., 2005). Isolate IMI330189 (hereafter called ‘189’) is a well characterised fungal entomopathogen (Driver et al., 2000) that has been the subject of intensive development as a biopesticide for locusts and grasshoppers (Lomer et al., 2001). Specific assays were developed for isolate IMI391510 and IMI330189 but not for ICIPE30. This latter isolate was used to test the accuracy of the two isolate-specific assays, and the utility of the “generic” (isolate-independent) fungal assay.

Application of fungal spores to the challenge pots was carried out according to the following protocol. Fungal spores were formulated in a mix of mineral oils (80% Isopar M:20% Ondina 22) similar to that described previously (Blanford et al., 2005) and the spore concentration adjusted to give 5 × 10^9^ spores/ml^−1^ (high dose), 1 × 10^9^ spores/ml^−1^ (medium dose) or 5 × 10^8^ spores/ml^−1^ (low dose). Spray applications employed a hand-held artist’s air brush which produced an aerosol of the spore formulation from a 25 ml glass jar attached to the spray nozzle. Each waxed cardboard challenge pot was opened and attached flat to the centre of the 1 m^2^ vertical spray zone within a laminar-flow hood. 20 ml of suspension was sprayed evenly from a distance of 25 cm across the entire spray zone providing the following theoretical conidial densities per dose: high, 1 × 10^7^ conidia/cm^2^; medium, 2 × 10^6^ conidia/cm^2^; and low, 1 × 10^6^ conidia/cm^2^. Pots were reassembled and mosquitoes then left in them for 6 h before being removed to untreated net cages where they were again provided with an *ad libitum* supply of glucose, kept at 25 °C and 80% RH and where they remained for the rest of the experiments’ duration.

### 2.3 DNA extraction

Quantification standards were obtained for all three fungal isolates by extracting DNA from 10^8^ of their respective conidia. Conidial suspensions in 0.05% Tween solution were counted using a hemocytometer and their numbers adjusted to 10^8^ ml^−1^. Aliquots (1 ml) were taken, the conidia pelleted by centrifugation, the tween solution removed and the pellets stored at −80 °C until required.

Mechanical disruption of conidia was achieved with a TissueLyser (Qiagen) under the following conditions. Altogether, 0.25 g of sterile 0.2 mm zirconium beads (OPS Diagnostics, LLC) and 0.25 g of sterile 0.8 mm silica beads (OPS Diagnostics, LLC) were added to each collection microtube bearing a conidial pellet and the sample dry ground for 1 min at 30 Hz. Microtubes were repositioned within the TissueLyser every 15 s to ensure uniformity of disruption for all samples. Four hundred microlitres of lysis solution from the DNeasy 96 Plant Kit™ (Qiagen) was then added to each tube and the samples ground for a further 1 min at 30 Hz, with the tube orientations changed every 15 s.

Extraction protocols utilizing different volumes and sizes of beads, different oscillation frequencies and time periods in the TissueLyser, wet or dry disruption and in the presence/absence of mosquitoes were all tested empirically (data not shown). The regime described was found to be optimal and linear for yield across seven orders of magnitude of conidia (10^8^–10^2^) and recovery was equivalent across the dynamic range in the presence or absence of a mosquito (Pearson correlation: *r*^2^ = 0.99, *p* < 0.001; intercept ≠ 0: *T* = 0.58, *p* = 0.57). Yields were also found to be equivalent or greater to those obtained by our previous “gold standard” methodology of grinding conidia with a pestle under liquid Nitrogen (data not shown) prior to DNA extraction. Under these conditions mosquitoes were thoroughly disrupted thereby exposing internal fungal burdens to the grinding action of the TissueLyser and the lysis solution.

Directly extracted conidial samples (from 10^8^ to 10^2^) yielded the same DNA concentrations as those obtained by the serial dilution of DNA from 10^8^ conidial extractions (Pearson correlation: *r*^2^ = 0.99, *p* < 0.001; intercept ≠ 0: *T* = 1.1, *p* = 0.3).

DNA was subsequently isolated from the disrupted and lysed samples using the DNeasy 96 Plant Kit™ (Qiagen) according to the manufacturers instructions, resuspended in 200 μl of elution buffer and stored at −80 °C. Challenged mosquitoes and samples of challenge pots were mechanically disrupted and the DNA collected using the same methodologies.

Quantitative standards for *P. chabaudi* were obtained by extracting DNA from a known number of infected murine red blood cells utilising the BloodPrep^®^ kit (Applied Biosystems) on the ABI Prism^®^ 6100 Nucleic Acid Prep Station according to manufacturer’s instructions, as described by Bell et al. (2006). DNA was eluted in a total volume of 200 μl, aliquoted, and stored at −80 °C.

### 2.4 Real-time quantitative PCR assays

Specific PCR primers and minor grove-binder (MGB) probes were designed using Primer Express^®^ (Applied Biosystems) software to develop four real-time quantitative PCR assays: a “generic” fungal assay for quantifying any of the fungi of interest; an assay “specific” for *B. bassiana* GHA-strain; an assay “specific” for *M. anisopliae* var. *acridium* isolate 189; and an assay for counting *Plasmodium* parasites.

Real-time quantitative PCRs were performed on an Applied Biosystems 7500 Fast Real-Time PCR System with an initial denaturation of 95 °C for 20 s followed by 40 cycles of denaturation at 95 °C for 3 s and annealing/extension at 60 °C for 30 s. Two microlitre of DNA was included in a 25 μl volume PCR reaction with the following components: 1.5 μl each of forward and reverse primer, both at a final concentration of 300 nM; 12.5 μl of 2 × PerfeC_T_a^TM^ qPCR FastMix^TM^, Low Rox; 1 μl of MGB probe at a final concentration of 200 nM and 6.5 μl of sterile water.

Absolute quantification of experimental samples was determined by comparing threshold cycle numbers against a standard curve. A series of quantification standards were generated from serial dilutions of a thawed *B. bassiana* DNA aliquot obtained from 10^8^ conidia. Three replicates of each DNA standard (covering six orders of magnitude from 10^7^ conidia to 10^2^ conidia) were included in each quantitative PCR run. 10^2^ conidia was considered the detection threshold of the fungal assays as only 1/100th (2 μl of 200 μl) of the total volume of DNA obtained was utilized in each qPCR: equivalent to the DNA extracted from a single conidium. Quantification is possible at levels below this due to the multiple copy number of the rRNA gene, but such counts are excessively influenced by pipetting variation.

### 2.5 Application of the *B. bassiana*-specific assay: the effect of challenge dose on *B. bassiana* replication within mosquitoes

Mosquitoes were placed into pots previously sprayed with a suspension of *B. bassiana* conidia at three different doses: high, 1 × 10^7^ conidia/cm^2^; medium, 2 × 10^6^ conidia/cm^2^; or low, 1 × 10^6^ conidia/cm^2^ as detailed above. Sub-samples of 20 live mosquitoes were removed from each challenge environment after 6 h (immediately post-exposure = conidial pick-up), and the remaining mosquitoes transferred to rearing cages (two cages per treatment with approximately 200 mosquitoes per cage). Sub-samples of these mosquitoes were then removed daily until day 6 post-challenge. Mosquitoes, killed by an overdose of chloroform, were placed in a bijoux (five individuals per container) containing a damp plug of cotton wool at its base. Bijouxs were kept horizontal so that the mosquitoes did not come into contact with the cotton wool plug and placed immediately into a −20 °C freezer until DNA extraction. Such storage has been previously shown to be stable for mosquito-borne DNAs (Bell and Ranford-Cartwright, 2004). A cohort of 20 mosquitoes were sub-sampled prior to the fungal challenge and quantified by both the “generic” fungal assay (to indicate background levels of fungi, such as *Aspergillus* sp., present in the rearing environment) and the *B. bassiana*-specific assay (to ensure no prior exposure to the challenge fungus). Fresh cadavers (less than 24 h since death) were collected on day 5 post-challenge. A further sub-sample of 30 live mosquitoes was also taken from the medium dose rearing cage on day 5 post-exposure, the mosquitoes dissected and identified as either visually-infected or visually-uninfected prior to the determination of their respective fungal burdens. Conidial densities actually present on the walls of challenge chambers (pots) were determined by counting the number of conidia (extraction and quantification methodologies as for mosquito material) present on eight 0.5 cm^2^ samples taken randomly across each pot after mosquitoes had been transferred to rearing cages.

## 3 Results

### 3.1 Real-time quantitative PCR assay development

Three qPCR assays were successfully developed for counting fungal genomes, thereby enabling a measure of the replication – based on nuclear division – of the target fungi. The assays were designed for the quantification of fungal burdens on/within mosquito hosts, but could equally be utilized for determining numbers in other hosts. Here quantification was based on conidial units (each unit being the equivalent of the DNA from a single conidia), but it could be performed in terms of ng DNA. It should be noted that standards derived from conidia require the use of conidia of the target fungi – the ploidy of conidia varying among different fungi.

All fungal assays were found to be linear over 7+ orders of magnitude and that developed for *P. chabaudi* was linear over 5+ orders of magnitude (upper testable limits restricted by parasite numbers attainable from infected murine blood and blood volumes tolerable to DNA extraction methodologies). Specificities of particular assays are provided below and in Table 1.

### 3.2 “Generic” fungal assay

The “generic” fungal assay targets a region of the 18S rRNA gene that, by inspection of Genbank, is highly conserved among fungi. Within the amplicon the two *Metarhizium* strains (132 bp) differ from *B. bassiana* (131 bp) in three point mutations: two transversions and an insertion/deletion. Sequence identity exists for all three fungi at primer/probe locations.

**Fig. 1:**
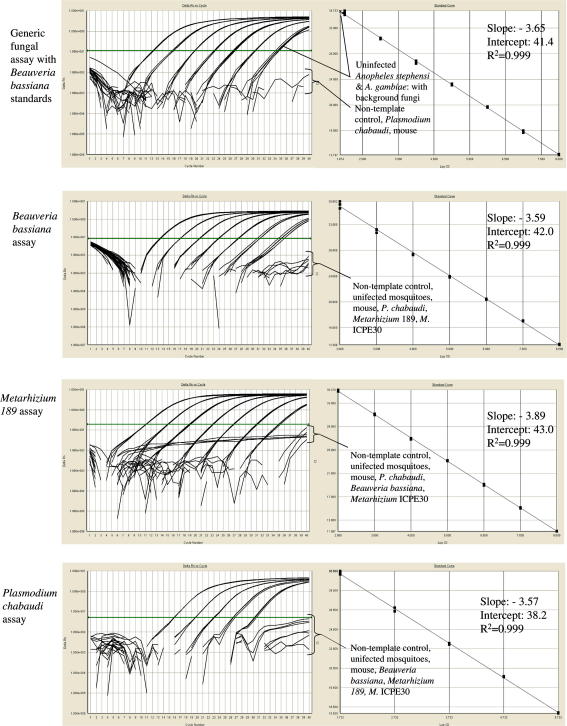
Assay amplification plots and standard curves. Left hand panels show real-time qPCR amplification plots: each set of parallel lines indicate a 10-fold dilution in DNA sample (known standards – for fungal assays from 10^8^ to 10^2^ conidia) from which the standard curves are derived (right hand panels) by plotting the cycle at which each standard enters into log-linear amplification (crosses the machine-determined threshold: solid horizontal line) against the known number of conidia present in that standard. Conidial numbers in unknown samples are determined from where their amplification plot crosses the threshold and is read from the standard curve. Non-amplified samples fail to generate an amplification plot and cross the threshold. A standard curve with a slope of −3.32 represents an assay with a hypothetical PCR efficiency of 100%.

The assay does not amplify mosquito, murine or *Plasmodium* DNA. It may be used to quantify any of the three fungi tested, although its deliberate lack of specificity will result in counts partially attributable to background fungi present in the natural/experimental environment (see Table 1, Fig. 1). Nevertheless, background counts typically recorded for unchallenged mosquitoes reared within our insectary were minimal: see *B. bassiana growth within challenged mosquitoes* section below.

The assay is highly reproducible: in 34 separate quantification runs, incorporating either *B. bassiana* or *Metarhizium* 189 standards, the mean efficiency of the qPCRs was 94.5% (mean (±SE) slope of standard curve over six orders of magnitude = −3.46 (±0.16), range = −3.27 to −3.71). The assay showed very high repeatability both within runs (0.99) and between runs (0.98) (Lessells and Boag, 1987), and has a reliable detection limit down to around 200-times the DNA from a single *B. bassiana* conidia in a PCR reaction or <0.01 pg DNA (determined by nano-spectrophotometer counts of standards, data not shown) .

### 3.3 Beauveria bassiana assay

The *B. bassiana* assay was specific to its target DNA, with no amplification of the two *Metarhizium* strains, background fungi, mosquito, murine or *Plasmodium* DNAs (see Table 1, Fig. 1). This assay amplifies part of the second Internal Transcribed Spacer (ITS2) region of the rRNA gene. It has a mean efficiency of 96% (slope of standard curve over six orders of magnitude = −3.42 (±0.11), range = −3.30 to −3.64), demonstrated very high repeatability both within runs (0.99) and between runs (0.98) and has a similar detection threshold to the generic fungal assay. Quantification of unknown samples performed with the generic fungal assay and *B. bassiana* assay were highly correlated (Pearson correlation: *r*^2^ = 0.99, *p* < 0.001; intercept ≠ 0: *T* = −1.3, *p* = 0.24).

### 3.4 M. anisopliae var. acridium (189) assay

The *Metarhizium* 189 assay also targets the variable ITS2 region of the rRNA gene. It was found to be specific, with no amplification of *B. bassiana*, *Metarhizium* ICPE30, background fungi, mosquito, murine or *Plasmodium* DNAs (see Table 1, Fig. 1). The detection threshold was equivalent to the two other fungal assays. Quantifications of unknown samples performed with the “generic” fungal assay and *Metarhizium* 189 assay were highly correlated (Pearson correlation: *r*^2^ = 0.99, *p* < 0.001; intercept ≠ 0: *T* = 0.00, *p* = 0.999) and very high repeatability both within runs (0.98) and between runs (0.97). The assay demonstrates a mean efficiency of 86% (slope of standard curve over six orders of magnitude = −3.71 (±0.14), range = −3.41 to −3.93).

The two assays specific to particular fungi (*B. bassiana* assay and *Metarhizium* 189 assay) may also be performed simultaneously in duplex qPCR reactions (specific probes labeled with different fluorophores) with no loss of sensitivity or specificity (data not shown).

### 3.5 Plasmodium assay

The *P. chabaudi* assay targets a region of the 18S rRNA gene that is highly conserved among *Plasmodium* spp. The assay successfully amplified DNA from a panel of eight distinct *P. chabaudi* clones (Mackinnon and Read, 1999; Bell et al., 2006), as well as *P. falciparum, P. malariae, P. berghei* and *P. yoelii*, all with equivalent qPCR efficiencies (data not shown). It does not amplify *B. bassiana*, either of the two *Metarhizium* strains, background fungi, mosquito or murine DNAs (see Table 1, Fig. 1).

The assay is highly reproducible: in 26 separate quantification runs (data not shown), incorporating either AS, AJ, AT, CB or CW *P. chabaudi*-clone standards (Bell et al., 2006), the mean efficiency of the quantitative PCRs was 94.9% (mean slope of standard curve over five orders of magnitude = −3.45, SE = 0.10, range = −3.30 to −3.62), with a detection limit <10 parasites/qPCR, which is equivalent to <200 parasites/μl blood.

### 3.6 B. bassiana growth within challenged mosquitoes

**Fig. 2:**
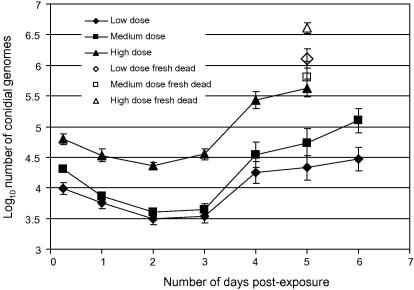
Number of *Beauveria bassiana* conidial units on/within mosquitoes with respect to time post-challenge. Symbols represent mean log_10_ number of conidial units at each sample time point for each challenge dose and vertical lines ±1SE.

**Fig. 3:**
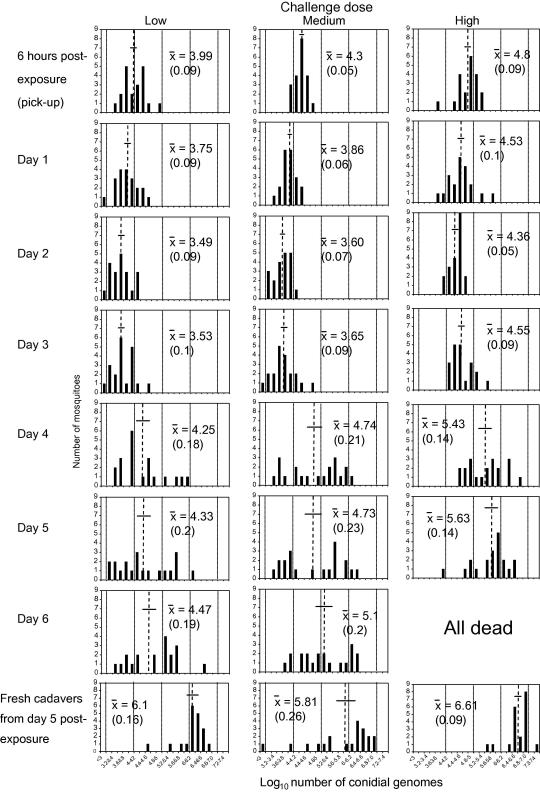
Number of *Beauveria bassiana* conidial units on/within mosquitoes with respect to time post-challenge. Solid bars represent the number of mosquitoes with a particular burden; dashed lines numerical means and horizontal lines (numbers in brackets) ±1SE.

The kinetics of fungal genome number in mosquitoes in response to three different challenge doses is shown in Figs. 2 and 3.

The “generic” fungal assay revealed unchallenged mosquitoes to have mean (±SE) background fungal counts of 347 (±37) conidial units (*B. bassiana* standards), whilst the *B. bassiana*-specific assay showed 12 of these 20 mosquitoes to be negative for *B. bassiana* genomes and 8 to bear only trace numbers (data not shown).

Actual conidial densities present on challenge pots are shown in Table 2. These differed 2.2-fold between the low and medium doses (in theory should be 2-fold) and 13.2-fold between the medium and high doses (in theory 5-fold). Nevertheless, conidial acquisition by mosquitoes exposed to the high dose was proportionately less than at the other two doses and there was a 1.9-fold difference in conidial pick-up between the low (ca. 1 × 10^4^) and medium (ca. 2 × 10^4^) doses and a 3.3-fold difference between the medium and high (ca. 6 × 10^4^) doses (see also Figs. 2 and 3). These differences in pick-up were significant between challenge doses (*F*_2,57_ = 27.2, *p* < 0.001). Fungal burdens on/within mosquitoes exposed to the two lower challenge doses did not differ significantly from each other at any subsequent sample point (*F*_1,39_ < 3.27, *p* > 0.08), whereas mean numbers of fungal genomes were significantly greater from mosquitoes exposed to the high dose at all sample points (*F*_1,39_ > 15.1, *p* < 0.002). By day 2 post-exposure mosquitoes from all challenge groups had significantly reduced burdens compared to their respective pick-up densities (*F*_1,39_ > 15.1, *p* < 0.001; see Fig. 2). Mean numbers of genomes then increased in all treatment groups between days 2 and 3 post-exposure, although not significantly so for any dose. Burdens increased significantly across all doses from day 3 to day 4 post-challenge (*F*_1,39_ > 12.2, *p* < 0.001) and continued to rise through to the end of the monitoring period at day 6 post-exposure (no mosquitoes remained alive in the high dose treatment on day 6), but there were no further significant increases in burdens. Fresh cadavers (collected at day 5 post-exposure) yielded significantly greater mean burdens than the respective mean densities from live mosquitoes (*F*_1,38_ > 4.8, *p* < 0.035).

**Fig. 4:**
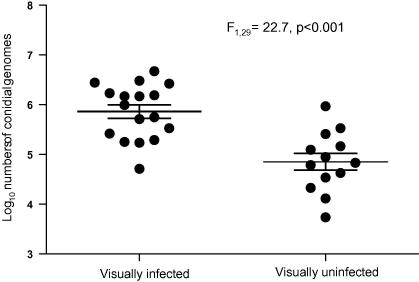
Relative burdens of visually-infected and visually-uninfected mosquitoes dissected on day 5 post-exposure. Symbols represent counts from individual mosquitoes; horizontal bars means and heavy horizontal bars ±1SE.

Fungal burdens in visually-infected and visually-uninfected mosquitoes differed significantly (*F*_1,29_ = 22.7, *p* < 0.001), but there was some margin of overlap between the two groups (see Fig. 4).

**Fig. 5:**
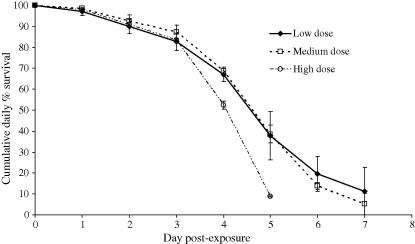
Mean cumulative daily percent survival of *Anopheles stephensi* exposed to either low (5 × 10^8^ spores/ml^−1^), medium (1 × 10^9^ spores/ml^−1^) or high (5 × 10^9^ spores/ml^−1^) formulations of *Beauveria bassiana* (see text for further application details). Survival was estimated as the number dying on a particular day as a percent of those alive at the end of the previous day. Survival does not reach zero as mosquitoes remaining alive on their last recorded survival day were sampled for PCR analysis. Mean and SEM are taken from two replicated cages per dose regime.

Fig. 5 shows the daily cumulative percentage survival for mosquitoes of each challenge dose.

## 4 Discussion

The TissueLyser^TM^ disruption method proved to be extremely effective and highly repeatable providing high yields of conidial/fungal DNA with no PCR-inhibiting contaminants resulting from the presence of the mosquito host. Castrillo et al. (2008) utilized 0.5 mm zirconia/silica beads and a Mini Bead Beater (Biospec Products) to successfully extract DNA from *B. bassiana* conidia and to these they added 0.7 mm zirconia beads for extractions from soil samples (see also Kuske et al., 1998). We also found 0.5 mm zirconia/silica beads to work well for conidial samples, but a combination of 0.2 mm zirconium beads and 0.8 mm silica beads (necessary for mosquito disruption) worked best for the combination of fungal conidia in the presence of a mosquito. Effective disruption of the mosquito host required an initial dry homogenization. This was not found to be detrimental to DNA yields from known numbers of conidia. DNA recovery was not improved with homogenization durations of greater than 2 min and indeed at 4 min yields were reduced, presumably due to shearing. In addition, the 96 well format of the TissueLyser^TM^ and DNeasy 96 Plant Kit^TM^ (Qiagen) enabled rapid throughput of materials with 192 samples easily processed in a day.

The qPCR assays reported here provide sensitive, target-specific, and robust quantification of fungal genomes.

Our *B. bassiana* assay amplifies a region of the multi-copy rRNA gene. The assay specific for *B. bassiana* GHA-strain developed by Castrillo et al. (2008) targeted the strain-specific sequence-characterised amplified region (SCAR) marker isolated by Castrillo et al. (2003) from an unknown gene of unknown function. These authors found their assay to have a detection threshold of 0.4 pg DNA. Unaware of their work at that time we also designed an assay to the same SCAR fragment, but we used different primers which generate a different amplicon (data not shown). Our assay demonstrated a very similar detection limit of approximately 0.2 pg DNA. This limit equates to between 10^3^ and 10^4^ conidia (DNA originating from >10 conidia present in a qPCR), some 10- to 100-fold less sensitive than the current ITS2-targeting assay – the differences in sensitivity presumably due to the respective copy numbers of the target genes. Nevertheless, whilst the current ITS2 assay is likely to amplify certain other *B. bassiana* isolates (not examined) Castrillo et al. (2008), tested theirs extensively and believed it to be specific to the strain GHA. Thus, each assay may be more suitable for particular applications dependant on the need for detection sensitivity or strain specificity. Detection thresholds of the “generic” fungal assay and *Metarhizium* 189-specific assay were equivalent to that of the *B. bassiana* ITS2 assay (<100 conidia or <0.01 ng DNA), with all assays targeting regions of the rRNA gene.

The spray application of a conidial suspension onto the opened surface of waxed pots within a laminar-flow hood was, unsurprisingly, found to be a not particularly efficient delivery method with between 90% (high dose) and 97% (low dose) of spores lost during the procedure. Conidial acquisition by mosquitoes placed within sprayed pots was found to be density dependent with 32% of the load from a single cm^2^ being “picked-up” by mosquitoes at pot densities of 3 × 10^4^ cm^−2^ (low dose), 26% at 7 × 10^4^ cm^−2^ (medium dose) and 7% at 9 × 10^5^ cm^−2^ (high dose). It may be that at the high dose proportionately fewer conidia are accessible to the mosquito due to a deeper carpet effect on the substratum, or that the numbers picked-up at the high dose are close to a limiting burden possible on the surface of a mosquito. It is also relevant that these were un-bloodfed mosquitoes and that just-fed settling mosquitoes are likely to be less active affecting conidial pick-up. Regardless of these variations the differences in acquisition magnitudes of the three challenge doses at 6 h post-exposure closely matched those sought with the high dose resulting in 3× the conidial pick-up of the middle dose that was in turn 2× that of the low dose. Such challenges with *B. bassiana* conidia resulted in surface burdens that were some 30-times (low dose) to 200-times (high dose) greater than background fungal counts on unchallenged mosquitoes (as determined by the “generic” fungal assay). One of the most important issues that can be addressed by qPCR in the biopesticide context is the determinants of spore dose contacted by target insects. The assays and techniques described here will enable the examination of different delivery methods and subsequent conidial pick-up by mosquitoes on different surfaces and under varying conditions. Given that elimination of background fungi will be impractical in most *Anopheles* settings, even in most insectaries, fungal-specific assays will probably be required for most dose-acquisition tests for delivery systems practical in the field.

We used the *B. bassiana*-specific assay to look at the kinetics of infections in mosquitoes exposed to *B. bassiana* at three challenge doses. The following picture of fungal infection in *Anopheles* emerged. Fungal counts went down by between 61% (high dose) and 78% (medium dose) over the first 2 days post-challenge, implying that at many conidia initially present on the mosquito surface were lost due to grooming or transfer from mosquitoes to mesh cage sides after introduction. The similarly low variance about the mean at 6 h, day 1 and day 2 post-challenge (in all 9 cases SEM <0.1 log units, see Fig. 3) indicates the uniformity of dose and retention of conidia among the treatment groups, and that any fungal replication over this period is unable to replace losses. Day 3 post-exposure saw increases in the mean burdens across all experimental groups although the number of fungal genomes had yet to re-attain pick-up levels. At this time point there was no significant difference between the cumulative mortality of the three treatment groups (Fig. 5) and only the high dose mosquitoes had any individuals infected with greater than 10^5^ conidial units (15%; Fig. 3). Between day 3 and day 4 post-exposure, marked fungal replication took place, with the mean number of *B. bassiana* genomes present within mosquitoes increasing more than 5-fold. There was also an increase in death rates across all challenge doses, although significantly more mortalities were now apparent among high dose mosquitoes (see Fig. 5). However, this mean increase in genome numbers represented marked nuclear division of fungi in certain mosquitoes, whilst in other hosts, fungal development remained static (compare panels for days 3 and 4 for all doses in Fig. 3). This disparity between individual hosts, regardless of challenge dose, is reflected in the increase of SEM to greater than 0.14 log units. The on-set of rapid genomic replication was related to dose with mosquitoes bearing greater than 10^5^ conidial units comprising 20% of the low dose cohort, 50% of the medium dose and 65% of the high dose; the two higher doses having 5% and 20% of individuals, respectively, with burdens in excess of 10^6^ units. Where there was a sudden on-set of substantial nuclear division, it was typically accompanied by physical fungal growth (hyphae) within the mosquito haemocoel, as evidenced by comparative counts in visually-infected and visually-uninfected hosts. Moreover, from the steep increase in host mortality (day 4 post-challenge), mean fungal burdens did not increase significantly. Thus, the marked increases in fungal replication in some mosquitoes must be shortly followed by host death, and hence the removal of these mosquitoes from the sample group of live mosquitoes. This conclusion is supported by our observation that the fungal burdens in all fresh cadavers were of similar magnitude to the very high counts recorded from a few live mosquitoes. Why the burst of fungal replication occurs much earlier in some mosquitoes than others, and whether the replication is a response to imminent death or the cause of it, remains to be determined. What is apparent is that challenge dose (within our experimental 6-fold range) does not appear to alter the growth form of *B. bassiana* infection in the anophelene host, but it does have a marked effect on the kinetics of the infection. Mosquitoes exposed to a higher conidial dose pick-up and continue to harbour greater mean fungal burdens with rapid genomic replication typically initiated earlier. This replication surge when initiated is accompanied by rapid hyphal growth and shortly followed by host death.

Having both a “generic” fungal assay and specific entomopathogen assays provides for flexibility in quantitative approach, whilst the ability to duplex assays further enhances experimental scope. We are currently employing assays to investigate what happens with cocktails of fungal entomopathogens, and whether variation in time to death associated with fungal strain and environmental conditions is associated with fungal replication kinetics within mosquitoes. This work, and other lines of research made possible by the qPCR assays described here, will contribute to optimization of fungal biopesticides against malaria and other vector-borne diseases.

## Figures and Tables

**Table 1:** Primer and probe sequences for real-time quantitative assays and assay specificities.

Assay and target locus	Primer and Probe sequences (5′–3′)	Amplicon size	Genbank accession no.	Assay specificity
“Generic” fungal assay	F: AGA TAC CGT CGT AGT CTT AAC CAT AAA CT;	131 bp	*B. bassiana*: AY245649	Amplifies: *B. bassiana*, *M. anisopliae* (var.
18S rRNA gene	R: TTC AGC CTT GCG ACC ATA CT;	132 bp	*M. anisopliae* var. *acridium*: AF487275	*acridium*) isolate 189, *M. anisopliae* (var. *anisopliae*) isolate ICPE30
	Probe: 6-FAM-CGT TCG GCA CCT TAC –MGB		*M. anisopliae* var. *anisopliae*: AF487273	Not amplify: mouse, mosquito, *P. chabaudi*
*Beauveria bassiana* assay	F: GCC GGC CCT GAA ATG G;	121 bp	AF345539	Amplifies: *B. bassiana*
ITSII rRNA gene	R: GAT TCG AGG TCA ACG TTC AGA AG;			Not amplify: *M. anisopliae* 189, *M. anisopliae*
	Probe: 6-FAM-ACA GCT CGC ACC GGA-MGB			ICPE30, background fungi, mouse, mosquito, *P. chabaudi*

*Metarhizium anisopliae*	F: GGA TCG GCG AAG CTT TTT TCA;	99 bp	EU307907	Amplifies: *M. anisopliae* 189
var. *acridium* (189)	R: CCC GTT GCG AGT GAG TTA CTA;			Not amplify: *M. anisopliae* IC30, *B. bassiana*,
ITSII rRNA gene	Probe: 6-FAM-CCG TCC CTT AAA TTT-MGB			background fungi, mouse, mosquito, *P. chabaudi*
*Plasmodium chabaudi*	F: TGT CAG AGG TGA AAT TCT TAG ATT TTC T;	88 bp	DQ241815	Amplifies: All tested *P. chabaudi* clones, *P.*
assay	R: ACT TTC GTT CTT GAT TAA TGG AAG TAT TT;			*falciparum*, *P. malariae*, *P. berghei* and *P. yoelii*
18S rRNA gene	Probe: 6-FAM-CAA ACA ACT GCG AAA GC			Not amplify: mouse, mosquito, fungi (named or background)

**Table 2:** Theoretical and actual *B. bassiana* conidial densities on challenge pots and conidial pick-up by mosquitoes for the three challenge doses. Figures beside horizontal arrows show proportion of spores that progressed between adjacent columns; figures beside vertical arrows show differences with the treatment group in the row above.

Theoretical conidial density on challenge pots		Actual conidial density on challenge pots (per cm^2^). *n* = 8 per dose		Conidial acquisition by mosquitoes (pick-up after 6 h exposure). *n* = 20 per dose
High:				
5 × 10^9^/ml	9%	Log_10_ = 5.95 (±0.06)	6.6% of cm^2^	Log_10_ = 4.77 (±0.09)
and 20 ml/m^2^	⇨	or 8.91 × 10^5^	⇨	or 5.9 × 10^4^
(or 1 × 10^7^ per cm^2^)				

⇧		⇧		⇧
Theroretical: 5x		Actual: 13.2x		Actual: 3.3x
Medium:	3.4%		26% of cm^2^	
1 × 10^9^/ml	⇨	Log_10_ = 4.83 (±0.07)	⇨	Log_10_ = 4.25 (±0.05)
and 20 ml/m^2^		or 6.76 × 10^4^		or 1.78 × 10^4^
(or 2 × 10^6^ per cm^2^)				
				
⇧		⇧		⇧
Theroretical: 2x		Actual: 2.2x		Actual: 1.9x
Low:	3.0%		32% of cm^2^	
5 × 10^8^/ml	⇨	Log_10_ = 4.48 (±0.11)	⇨	Log_10_ = 3.98 (±0.09)
and 20 ml/m^2^		or 3.02 × 10^4^		or 9.55 × 10^3^
(or 1 × 10^6^ per cm^2^)				
